# Supplementary material for: Modeling glioblastoma heterogeneity as a dynamic network of cell states
Source: Mol Syst Biol. 2021 Sep 16;17(9):e10105. doi: 10.15252/msb.202010105 (PMC8444284; doi:10.15252/msb.202010105)
Supplement: Supplementary file 6 — Source Data for Figure 5 [file MSB-17-e10105-s004.zip › Figure5A_sourcedata/GSEA_3017/nosigdb_stateF.GseaPreranked.1622795015644/gsea_report_for_na_pos_1622795015644.html]

Report for na\_pos 1622795015644 [GSEA]

| GS  follow link to MSigDB | GS DETAILS | SIZE | ES | NES | NOM p-val | FDR q-val | FWER p-val | RANK AT MAX | LEADING EDGE || 1 | RICHARDS\_ZHONG\_NATURE\_2018\_ASTROCYTES\_UPREG | Details ... | 165 | 0.52 | 3.61 | 0.000 | 0.000 | 0.000 | 701 | tags=65%, list=31%, signal=88% |
| 2 | SUVA\_AC\_PMID\_31327527 | Details ... | 32 | 0.77 | 3.56 | 0.000 | 0.000 | 0.000 | 93 | tags=59%, list=4%, signal=61% |
| 3 | RICHARDS\_NEFTEL\_CELL\_2019\_AC | Details ... | 32 | 0.77 | 3.55 | 0.000 | 0.000 | 0.000 | 93 | tags=59%, list=4%, signal=61% |
| 4 | RICHARDS\_NOWAKOWSKI\_SCIENCE\_2017\_ASTROCYTE\_UPREG | Details ... | 267 | 0.47 | 3.47 | 0.000 | 0.000 | 0.000 | 637 | tags=54%, list=28%, signal=67% |
| 5 | RICHARDS\_NOWAKOWSKI\_SCIENCE\_2017\_ORG\_UPREG | Details ... | 491 | 0.41 | 3.28 | 0.000 | 0.000 | 0.000 | 632 | tags=48%, list=28%, signal=52% |
| 6 | LARSSON\_STATE\_6 | Details ... | 63 | 0.59 | 3.27 | 0.000 | 0.000 | 0.000 | 609 | tags=70%, list=27%, signal=93% |
| 7 | RICHARDS\_CAHOY\_JNEUROSCI\_2015\_ASTRO\_IN\_VIVO | Details ... | 416 | 0.39 | 3.07 | 0.000 | 0.000 | 0.000 | 845 | tags=58%, list=37%, signal=76% |
| 8 | RICHARDS\_ZHONG\_NATURE\_2018\_MICROGLIA\_UPREG | Details ... | 87 | 0.50 | 2.97 | 0.000 | 0.000 | 0.000 | 488 | tags=55%, list=22%, signal=68% |
| 9 | GAROFANO\_IAVARONE\_MTC\_SUBTYPE | Details ... | 80 | 0.50 | 2.97 | 0.000 | 0.000 | 0.000 | 651 | tags=61%, list=29%, signal=83% |
| 10 | RICHARDS\_ZHONG\_NATURE\_2018\_OPC\_UPREG | Details ... | 50 | 0.56 | 2.95 | 0.000 | 0.000 | 0.000 | 677 | tags=74%, list=30%, signal=103% |
| 11 | TISIG\_PMID\_32649888 | Details ... | 37 | 0.61 | 2.93 | 0.000 | 0.000 | 0.000 | 582 | tags=73%, list=26%, signal=97% |
| 12 | VANGRONINGEN\_MES | Details ... | 142 | 0.43 | 2.88 | 0.000 | 0.000 | 0.000 | 979 | tags=76%, list=43%, signal=126% |
| 13 | RICHARDS\_NOWAKOWSKI\_SCIENCE\_2017\_MURAL\_UPREG | Details ... | 210 | 0.40 | 2.88 | 0.000 | 0.000 | 0.000 | 834 | tags=63%, list=37%, signal=90% |
| 14 | RICHARDS\_NOWAKOWSKI\_SCIENCE\_2017\_OPC\_UPREG | Details ... | 153 | 0.42 | 2.88 | 0.000 | 0.000 | 0.000 | 681 | tags=56%, list=30%, signal=75% |
| 15 | OLSEN\_SCPLIKE | Details ... | 48 | 0.55 | 2.86 | 0.000 | 0.000 | 0.000 | 349 | tags=48%, list=15%, signal=55% |
| 16 | RICHARDS\_NOWAKOWSKI\_SCIENCE\_2017\_VRG\_UPREG | Details ... | 183 | 0.41 | 2.86 | 0.000 | 0.000 | 0.000 | 621 | tags=50%, list=28%, signal=64% |
| 17 | DOROSZKO\_U3180MG\_CLUSTER\_5 | Details ... | 23 | 0.69 | 2.83 | 0.000 | 0.000 | 0.000 | 366 | tags=70%, list=16%, signal=82% |
| 18 | ALLEN\_113\_ASTROL1FGFR3FOS | Details ... | 19 | 0.74 | 2.83 | 0.000 | 0.000 | 0.000 | 93 | tags=53%, list=4%, signal=54% |
| 19 | RICHARDS\_NOWAKOWSKI\_SCIENCE\_2017\_MICROGLIA\_UPREG | Details ... | 213 | 0.39 | 2.82 | 0.000 | 0.000 | 0.000 | 514 | tags=43%, list=23%, signal=50% |
| 20 | DOROSZKO\_U3013MG\_CLUSTER\_3 | Details ... | 65 | 0.49 | 2.80 | 0.000 | 0.000 | 0.000 | 562 | tags=55%, list=25%, signal=72% |
| 21 | GANDAL\_SCHIZOPHRENIA\_UP\_PMID29439242 |  | 63 | 0.50 | 2.79 | 0.000 | 0.000 | 0.000 | 241 | tags=40%, list=11%, signal=43% |
| 22 | SUVA\_MES2\_PMID\_31327527 |  | 17 | 0.72 | 2.77 | 0.000 | 0.000 | 0.000 | 228 | tags=65%, list=10%, signal=71% |
| 23 | RICHARDS\_CAHOY\_JNEUROSCI\_2008\_ASTROCYTE |  | 445 | 0.34 | 2.76 | 0.000 | 0.000 | 0.000 | 639 | tags=46%, list=28%, signal=52% |
| 24 | DOROSZKO\_U3013MG\_CLUSTER\_1 |  | 21 | 0.68 | 2.76 | 0.000 | 0.000 | 0.000 | 367 | tags=71%, list=16%, signal=85% |
| 25 | OLSEN\_MESENCHYMAL |  | 40 | 0.54 | 2.71 | 0.000 | 0.000 | 0.000 | 593 | tags=63%, list=26%, signal=83% |
| 26 | NB\_KI\_CLUSTER18 |  | 42 | 0.54 | 2.69 | 0.000 | 0.000 | 0.000 | 347 | tags=43%, list=15%, signal=50% |
| 27 | ALLEN\_112\_ASTROL1FGFR3MT1G |  | 19 | 0.69 | 2.68 | 0.000 | 0.000 | 0.000 | 138 | tags=53%, list=6%, signal=56% |
| 28 | POLLEN\_OUTERRADIALGLIA\_PMID\_26406371 |  | 31 | 0.59 | 2.68 | 0.000 | 0.000 | 0.000 | 718 | tags=84%, list=32%, signal=121% |
| 29 | OUTERRADIALGLIA |  | 31 | 0.59 | 2.68 | 0.000 | 0.000 | 0.000 | 718 | tags=84%, list=32%, signal=121% |
| 30 | RICHARDS\_NEFTEL\_CELL\_2019\_MES2 |  | 17 | 0.72 | 2.66 | 0.000 | 0.000 | 0.000 | 228 | tags=65%, list=10%, signal=71% |
| 31 | RICHARDS\_CAHOY\_JNEUROSCI\_2011\_ASTRO\_MATURE |  | 169 | 0.38 | 2.64 | 0.000 | 0.000 | 0.000 | 674 | tags=51%, list=30%, signal=68% |
| 32 | DOROSZKO\_U3054MG\_CLUSTER\_3 |  | 77 | 0.44 | 2.60 | 0.000 | 0.000 | 0.000 | 604 | tags=55%, list=27%, signal=72% |
| 33 | DOROSZKO\_U3054MG\_CLUSTER\_2 |  | 111 | 0.40 | 2.56 | 0.000 | 0.000 | 0.000 | 853 | tags=62%, list=38%, signal=95% |
| 34 | GAROFANO\_IAVARONE\_GPM\_SUBTYPE |  | 247 | 0.34 | 2.52 | 0.000 | 0.000 | 0.001 | 635 | tags=45%, list=28%, signal=56% |
| 35 | IVY\_IT |  | 32 | 0.55 | 2.52 | 0.000 | 0.000 | 0.001 | 731 | tags=75%, list=32%, signal=109% |
| 36 | ALLEN\_111\_ASTROL1\_6FGFR3ETNPPL |  | 19 | 0.64 | 2.52 | 0.000 | 0.000 | 0.001 | 93 | tags=42%, list=4%, signal=44% |
| 37 | DOROSZKO\_U3180MG\_CLUSTER\_2 |  | 165 | 0.36 | 2.49 | 0.000 | 0.000 | 0.001 | 653 | tags=48%, list=29%, signal=62% |
| 38 | GANDAL\_BIPOLAR\_UP\_PMID29439242 |  | 44 | 0.49 | 2.48 | 0.000 | 0.000 | 0.001 | 527 | tags=57%, list=23%, signal=73% |
| 39 | RICHARDS\_NOWAKOWSKI\_SCIENCE\_2017\_EN\_PFC1\_UPREG |  | 271 | 0.33 | 2.47 | 0.000 | 0.000 | 0.001 | 1201 | tags=78%, list=53%, signal=147% |
| 40 | GANDAL\_AUTISM\_UP\_PMID29439242 |  | 39 | 0.49 | 2.46 | 0.000 | 0.000 | 0.001 | 234 | tags=36%, list=10%, signal=39% |
| 41 | RICHARDS\_NOWAKOWSKI\_SCIENCE\_2017\_ENDOTHELIAL\_UPREG |  | 397 | 0.31 | 2.45 | 0.000 | 0.000 | 0.001 | 632 | tags=42%, list=28%, signal=48% |
| 42 | LARSSON\_STATE\_2 |  | 39 | 0.50 | 2.44 | 0.000 | 0.000 | 0.001 | 373 | tags=46%, list=17%, signal=54% |
| 43 | SUVA\_MES1\_PMID\_31327527 |  | 22 | 0.59 | 2.40 | 0.000 | 0.000 | 0.001 | 249 | tags=50%, list=11%, signal=56% |
| 44 | GAROFANO\_IAVARONE\_NEU\_SUBTYPE |  | 65 | 0.42 | 2.39 | 0.000 | 0.000 | 0.001 | 724 | tags=60%, list=32%, signal=86% |
| 45 | RICHARDS\_NOWAKOWSKI\_SCIENCE\_2017\_TRG\_UPREG |  | 379 | 0.30 | 2.38 | 0.000 | 0.000 | 0.001 | 620 | tags=41%, list=28%, signal=47% |
| 46 | RICHARDS\_NEFTEL\_CELL\_2019\_MES1 |  | 22 | 0.59 | 2.38 | 0.000 | 0.000 | 0.001 | 249 | tags=50%, list=11%, signal=56% |
| 47 | RICHARDS\_NOWAKOWSKI\_SCIENCE\_2017\_U1\_UPREG |  | 314 | 0.30 | 2.37 | 0.000 | 0.000 | 0.002 | 882 | tags=56%, list=39%, signal=79% |
| 48 | IVY\_CTPAN |  | 46 | 0.46 | 2.35 | 0.000 | 0.000 | 0.002 | 613 | tags=59%, list=27%, signal=79% |
| 49 | RICHARDS\_CAHOY\_JNEUROSCI\_2014\_MOG\_POS |  | 171 | 0.34 | 2.34 | 0.000 | 0.000 | 0.002 | 792 | tags=56%, list=35%, signal=80% |
| 50 | PARADA\_T2\_DN\_26461091 |  | 16 | 0.64 | 2.32 | 0.000 | 0.000 | 0.003 | 826 | tags=100%, list=37%, signal=157% |
| 51 | RICHARDS\_VERHAAK\_CANCERCELL\_2010\_CLASSICAL |  | 50 | 0.44 | 2.28 | 0.000 | 0.000 | 0.007 | 645 | tags=60%, list=29%, signal=82% |
| 52 | NB\_KI\_CLUSTER23 |  | 89 | 0.37 | 2.26 | 0.000 | 0.000 | 0.007 | 1077 | tags=78%, list=48%, signal=143% |
| 53 | DOROSZKO\_U3054MG\_CLUSTER\_10 |  | 73 | 0.39 | 2.25 | 0.000 | 0.000 | 0.008 | 906 | tags=68%, list=40%, signal=111% |
| 54 | RICHARDS\_NOWAKOWSKI\_SCIENCE\_2017\_MGE\_RG1\_UPREG |  | 126 | 0.34 | 2.23 | 0.000 | 0.000 | 0.008 | 1004 | tags=68%, list=45%, signal=116% |
| 55 | NB\_KI\_CLUSTER13 |  | 50 | 0.42 | 2.22 | 0.000 | 0.000 | 0.008 | 649 | tags=54%, list=29%, signal=74% |
| 56 | NB\_KI\_CLUSTER17 |  | 59 | 0.41 | 2.22 | 0.000 | 0.000 | 0.008 | 947 | tags=76%, list=42%, signal=128% |
| 57 | RICHARDS\_NOWAKOWSKI\_SCIENCE\_2017\_EN\_V1\_3\_UPREG |  | 234 | 0.30 | 2.21 | 0.000 | 0.000 | 0.012 | 737 | tags=49%, list=33%, signal=65% |
| 58 | RICHARDS\_NOWAKOWSKI\_SCIENCE\_2017\_EN\_PFC3\_UPREG |  | 82 | 0.37 | 2.18 | 0.000 | 0.000 | 0.013 | 428 | tags=40%, list=19%, signal=48% |
| 59 | NB\_KI\_CLUSTER2 |  | 85 | 0.35 | 2.14 | 0.000 | 0.001 | 0.027 | 1046 | tags=72%, list=46%, signal=129% |
| 60 | RICHARDS\_CAHOY\_JNEUROSCI\_2013\_OL\_MYEL |  | 272 | 0.28 | 2.12 | 0.000 | 0.001 | 0.033 | 800 | tags=51%, list=35%, signal=69% |
| 61 | RICHARDS\_VERHAAK\_CANCERCELL\_2010\_MESENCHYMAL |  | 30 | 0.46 | 2.09 | 0.002 | 0.001 | 0.046 | 692 | tags=63%, list=31%, signal=90% |
| 62 | RICHARDS\_NOWAKOWSKI\_SCIENCE\_2017\_EN\_PFC2\_UPREG |  | 265 | 0.28 | 2.07 | 0.000 | 0.001 | 0.053 | 713 | tags=46%, list=32%, signal=59% |
| 63 | WANG\_CLASSICAL\_PMID\_28697342 |  | 19 | 0.52 | 2.07 | 0.002 | 0.001 | 0.053 | 887 | tags=84%, list=39%, signal=138% |
| 64 | DOROSZKO\_U3054MG\_CLUSTER\_5 |  | 43 | 0.41 | 2.07 | 0.002 | 0.001 | 0.057 | 608 | tags=51%, list=27%, signal=69% |
| 65 | GANDAL\_IBD\_UP\_PMID29439242 |  | 26 | 0.47 | 2.01 | 0.002 | 0.002 | 0.103 | 692 | tags=62%, list=31%, signal=88% |
| 66 | RICHARDS\_NEFTEL\_CELL\_2019\_OPC |  | 29 | 0.45 | 2.00 | 0.002 | 0.002 | 0.114 | 636 | tags=66%, list=28%, signal=90% |
| 67 | RICHARDS\_NOWAKOWSKI\_SCIENCE\_2017\_EN\_V1\_1\_UPREG |  | 250 | 0.27 | 1.99 | 0.000 | 0.002 | 0.119 | 717 | tags=46%, list=32%, signal=60% |
| 68 | SUVA\_OPC\_PMID\_31327527 |  | 29 | 0.45 | 1.99 | 0.008 | 0.002 | 0.119 | 636 | tags=66%, list=28%, signal=90% |
| 69 | PARADA\_T1\_DN\_26461091 |  | 17 | 0.53 | 1.99 | 0.009 | 0.002 | 0.123 | 826 | tags=82%, list=37%, signal=129% |
| 70 | IVY\_LE |  | 21 | 0.47 | 1.92 | 0.005 | 0.004 | 0.196 | 873 | tags=76%, list=39%, signal=123% |
| 71 | SINGH\_SOX2\_DOWNREGULATED\_TRANSCRIPTS\_PMID\_28122245 |  | 31 | 0.42 | 1.89 | 0.006 | 0.005 | 0.250 | 261 | tags=32%, list=12%, signal=36% |
| 72 | NB\_KI\_CLUSTER15 |  | 27 | 0.41 | 1.85 | 0.007 | 0.006 | 0.307 | 925 | tags=74%, list=41%, signal=124% |
| 73 | NB\_KI\_CLUSTER11 |  | 15 | 0.51 | 1.82 | 0.009 | 0.007 | 0.362 | 228 | tags=40%, list=10%, signal=44% |
| 74 | RICHARDS\_NOWAKOWSKI\_SCIENCE\_2017\_NIN1\_UPREG |  | 44 | 0.35 | 1.82 | 0.004 | 0.008 | 0.381 | 810 | tags=61%, list=36%, signal=94% |
| 75 | GANDAL\_ALCOHOLADDICTION\_DN\_PMID29439242 |  | 30 | 0.40 | 1.81 | 0.009 | 0.008 | 0.392 | 537 | tags=53%, list=24%, signal=69% |
| 76 | RICHARDS\_NOWAKOWSKI\_SCIENCE\_2017\_GLYC\_UPREG |  | 198 | 0.26 | 1.81 | 0.000 | 0.008 | 0.394 | 452 | tags=30%, list=20%, signal=34% |
| 77 | NB\_KI\_CLUSTER14 |  | 69 | 0.32 | 1.80 | 0.005 | 0.008 | 0.410 | 914 | tags=62%, list=41%, signal=102% |
| 78 | DOROSZKO\_U3054MG\_CLUSTER\_0 |  | 22 | 0.44 | 1.80 | 0.010 | 0.009 | 0.426 | 677 | tags=64%, list=30%, signal=90% |
| 79 | RICHARDS\_NOWAKOWSKI\_SCIENCE\_2017\_CHOROID\_UPREG |  | 164 | 0.26 | 1.79 | 0.002 | 0.009 | 0.436 | 495 | tags=34%, list=22%, signal=40% |
| 80 | DOROSZKO\_PERIVASCULAR\_INVASION |  | 19 | 0.46 | 1.78 | 0.009 | 0.010 | 0.471 | 834 | tags=74%, list=37%, signal=116% |
| 81 | RICHARDS\_CAHOY\_JNEUROSCI\_2009\_NEURON |  | 220 | 0.24 | 1.77 | 0.000 | 0.010 | 0.492 | 759 | tags=45%, list=34%, signal=61% |
| 82 | LARSSON\_STATE\_5 |  | 37 | 0.36 | 1.73 | 0.009 | 0.013 | 0.592 | 222 | tags=30%, list=10%, signal=32% |
| 83 | DOROSZKO\_U3013MG\_CLUSTER\_0 |  | 17 | 0.45 | 1.70 | 0.022 | 0.016 | 0.654 | 769 | tags=71%, list=34%, signal=106% |
| 84 | ALMSTEDT\_VGR\_DIFF |  | 258 | 0.22 | 1.64 | 0.000 | 0.025 | 0.814 | 504 | tags=33%, list=22%, signal=38% |
| 85 | RICHARDS\_NOWAKOWSKI\_SCIENCE\_2017\_EN\_V1\_2\_UPREG |  | 197 | 0.22 | 1.58 | 0.011 | 0.034 | 0.898 | 992 | tags=57%, list=44%, signal=93% |
| 86 | GANDAL\_ALCOHOLADDICTION\_UP\_PMID29439242 |  | 39 | 0.33 | 1.58 | 0.041 | 0.034 | 0.903 | 234 | tags=26%, list=10%, signal=28% |
| 87 | ALMSTEDT\_ALK |  | 28 | 0.36 | 1.57 | 0.047 | 0.036 | 0.917 | 443 | tags=32%, list=20%, signal=40% |
| 88 | RICHARDS\_NOWAKOWSKI\_SCIENCE\_2017\_IN\_CTX\_MGE2\_UPREG |  | 64 | 0.28 | 1.56 | 0.038 | 0.039 | 0.936 | 912 | tags=61%, list=40%, signal=99% |
| 89 | RICHARDS\_NOWAKOWSKI\_SCIENCE\_2017\_NEN\_EARLY2\_UPREG |  | 104 | 0.24 | 1.52 | 0.026 | 0.048 | 0.972 | 966 | tags=55%, list=43%, signal=91% |
| 90 | IVY\_CTMVP |  | 21 | 0.37 | 1.51 | 0.067 | 0.051 | 0.982 | 830 | tags=62%, list=37%, signal=97% |
| 91 | RICHARDS\_NOWAKOWSKI\_SCIENCE\_2017\_IPC\_NEN1\_UPREG |  | 79 | 0.24 | 1.44 | 0.051 | 0.076 | 0.997 | 1239 | tags=75%, list=55%, signal=160% |
| 92 | ANASTASSIOU\_MULTICANCER\_INVASIVENESS\_PMID\_22208948 |  | 15 | 0.39 | 1.43 | 0.099 | 0.076 | 0.997 | 593 | tags=53%, list=26%, signal=72% |
| 93 | RICHARDS\_NEFTEL\_CELL\_2019\_NPC1 |  | 17 | 0.35 | 1.32 | 0.158 | 0.138 | 1.000 | 810 | tags=65%, list=36%, signal=100% |
| 94 | RICHARDS\_NOWAKOWSKI\_SCIENCE\_2017\_IN\_CTX\_MGE1\_UPREG |  | 76 | 0.22 | 1.29 | 0.156 | 0.162 | 1.000 | 408 | tags=28%, list=18%, signal=33% |
| 95 | SUVA\_NPC1\_PMID\_31327527 |  | 17 | 0.35 | 1.28 | 0.189 | 0.173 | 1.000 | 810 | tags=65%, list=36%, signal=100% |
| 96 | RICHARDS\_NOWAKOWSKI\_SCIENCE\_2017\_IPC\_NEN3\_UPREG |  | 74 | 0.21 | 1.23 | 0.180 | 0.211 | 1.000 | 878 | tags=51%, list=39%, signal=81% |
| 97 | RICHARDS\_NOWAKOWSKI\_SCIENCE\_2017\_IN\_CTX\_CGE2\_UPREG |  | 79 | 0.21 | 1.21 | 0.187 | 0.229 | 1.000 | 437 | tags=28%, list=19%, signal=33% |
| 98 | RICHARDS\_CAHOY\_JNEUROSCI\_2008\_OLIGODENDROCYTE |  | 294 | 0.15 | 1.16 | 0.191 | 0.291 | 1.000 | 723 | tags=37%, list=32%, signal=48% |
| 99 | RICHARDS\_NOWAKOWSKI\_SCIENCE\_2017\_NIN3\_UPREG |  | 40 | 0.24 | 1.16 | 0.288 | 0.296 | 1.000 | 461 | tags=30%, list=20%, signal=37% |
| 100 | KRONA\_ROSEN\_WHITE\_MATTER\_INVASION |  | 69 | 0.20 | 1.15 | 0.261 | 0.296 | 1.000 | 540 | tags=38%, list=24%, signal=48% |
| 101 | ALMSTEDT\_11Q\_MSIGDB |  | 79 | 0.20 | 1.15 | 0.276 | 0.298 | 1.000 | 469 | tags=29%, list=21%, signal=35% |
| 102 | NB\_KI\_CLUSTER6 |  | 75 | 0.19 | 1.13 | 0.278 | 0.322 | 1.000 | 648 | tags=35%, list=29%, signal=47% |
| 103 | RICHARDS\_VERHAAK\_CANCERCELL\_2010\_PRONEURAL |  | 50 | 0.20 | 1.05 | 0.410 | 0.447 | 1.000 | 518 | tags=34%, list=23%, signal=43% |
| 104 | RICHARDS\_NOWAKOWSKI\_SCIENCE\_2017\_NIN4\_UPREG |  | 31 | 0.22 | 0.99 | 0.475 | 0.540 | 1.000 | 340 | tags=23%, list=15%, signal=26% |
| 105 | RICHARDS\_NOWAKOWSKI\_SCIENCE\_2017\_IN\_STR\_UPREG |  | 37 | 0.21 | 0.99 | 0.447 | 0.542 | 1.000 | 476 | tags=30%, list=21%, signal=37% |
| 106 | RICHARDS\_NOWAKOWSKI\_SCIENCE\_2017\_IN\_CTX\_CGE1\_UPREG |  | 73 | 0.17 | 0.97 | 0.496 | 0.565 | 1.000 | 298 | tags=18%, list=13%, signal=20% |
| 107 | NB\_KI\_CLUSTER4 |  | 32 | 0.20 | 0.93 | 0.562 | 0.641 | 1.000 | 352 | tags=25%, list=16%, signal=29% |
| 108 | DOROSZKO\_U3054MG\_CLUSTER\_8 |  | 129 | 0.14 | 0.92 | 0.613 | 0.644 | 1.000 | 520 | tags=23%, list=23%, signal=28% |
| 109 | RICHARDS\_NOWAKOWSKI\_SCIENCE\_2017\_U4\_UPREG |  | 334 | 0.12 | 0.92 | 0.658 | 0.645 | 1.000 | 495 | tags=23%, list=22%, signal=25% |
| 110 | NB\_KI\_CLUSTER16 |  | 26 | 0.22 | 0.90 | 0.598 | 0.668 | 1.000 | 830 | tags=50%, list=37%, signal=78% |
| 111 | RICHARDS\_NOWAKOWSKI\_SCIENCE\_2017\_NEN\_LATE\_UPREG |  | 95 | 0.14 | 0.85 | 0.700 | 0.756 | 1.000 | 1086 | tags=54%, list=48%, signal=99% |
| 112 | RICHARDS\_NOWAKOWSKI\_SCIENCE\_2017\_NIN2\_UPREG |  | 126 | 0.13 | 0.84 | 0.758 | 0.758 | 1.000 | 780 | tags=39%, list=35%, signal=56% |
| 113 | GANDAL\_DEPRESSION\_DN\_PMID29439242 |  | 17 | 0.22 | 0.83 | 0.679 | 0.777 | 1.000 | 923 | tags=59%, list=41%, signal=99% |
| 114 | RICHARDS\_NOWAKOWSKI\_SCIENCE\_2017\_NIN5\_UPREG |  | 80 | 0.14 | 0.80 | 0.759 | 0.810 | 1.000 | 611 | tags=33%, list=27%, signal=43% |
| 115 | DOROSZKO\_U3054MG\_CLUSTER\_9 |  | 112 | 0.11 | 0.69 | 0.922 | 0.925 | 1.000 | 490 | tags=21%, list=22%, signal=26% |
| 116 | NB\_KI\_CLUSTER8 |  | 62 | 0.11 | 0.59 | 0.951 | 0.973 | 1.000 | 475 | tags=23%, list=21%, signal=28% |
| 117 | RICHARDS\_NOWAKOWSKI\_SCIENCE\_2017\_IPC\_NEN1\_UPREG\_1 |  | 41 | 0.10 | 0.50 | 0.969 | 0.994 | 1.000 | 107 | tags=7%, list=5%, signal=8% |
| 118 | NB\_KI\_CLUSTER22 |  | 31 | 0.11 | 0.49 | 0.982 | 0.989 | 1.000 | 892 | tags=45%, list=40%, signal=74% |
Table: Gene sets enriched in phenotype **na **[plain text format]****
